# Revealing the Variation and Stability of Bacterial Communities in Tomato Rhizosphere Microbiota

**DOI:** 10.3390/microorganisms8020170

**Published:** 2020-01-25

**Authors:** Zhiqiang Cheng, Shaonan Lei, Ye Li, Wei Huang, Rongqin Ma, Juan Xiong, Ting Zhang, Lingyue Jin, Hafeez ul Haq, Xiaohong Xu, Baoyu Tian

**Affiliations:** 1 Provincial University Key Laboratory of Cellular Stress Response and Metabolic Regulation, College of Life Sciences, Fujian Normal University, Fuzhou 350108, Fujian, China; ChengZQJX@163.com (Z.C.); Leisn125@163.com (S.L.); ly111094@163.com (Y.L.); mrq15880200656@163.com (R.M.); xiongjuanJX@163.com (J.X.); 13070706374@163.com (T.Z.); jly9647@163.com (L.J.); hafeez4616@gmail.com (H.u.H.); 2 Institute of Quality Standards and Testing Technology for Agro-Products, Fujian Academy of Agricultural Sciences, Fuzhou 350003, Fujian, China; lifehuangwei@aliyun.com; 3 Library, Fujian Normal University, Fuzhou 350108, Fujian, China; xuxh008@aliyun.com

**Keywords:** tomato, rhizosphere microbiota, host genotype (cultivar), soils, shaping mechanism

## Abstract

Microorganisms that colonize the plant rhizosphere can contribute to plant health, growth and productivity. Although the importance of the rhizosphere microbiome is known, we know little about the underlying mechanisms that drive microbiome assembly and composition. In this study, the variation, assembly and composition of rhizobacterial communities in 11 tomato cultivars, combined with one cultivar in seven different sources of soil and growing substrate, were systematically investigated. The tomato rhizosphere microbiota was dominated by bacteria from the phyla Proteobacteria, Bacteroidetes, and Acidobacteria, mainly comprising Rhizobiales, Xanthomonadales, Burkholderiales, Nitrosomonadales, Myxococcales, Sphingobacteriales, Cytophagales and Acidobacteria subgroups. The bacterial community in the rhizosphere microbiota of the samples in the cultivar experiment mostly overlapped with that of tomato cultivar MG, which was grown in five natural field soils, DM, JX, HQ, QS and XC. The results supported the hypothesis that tomato harbors largely conserved communities and compositions of rhizosphere microbiota that remains consistent in different cultivars of tomato and even in tomato cultivar grown in five natural field soils. However, significant differences in OTU richness (*p* < 0.0001) and bacterial diversity (*p* = 0.0014 < 0.01) were observed among the 7 different sources of soil and growing substrate. Two artificial commercial nutrient soils, HF and CF, resulted in a distinct tomato rhizosphere microbiota in terms of assembly and core community compared with that observed in natural field soils. PERMANOVA of beta diversity based on the combined data from the cultivar and soil experiments demonstrated that soil (growing substrate) and plant genotype (cultivar) had significant impacts on the rhizosphere microbial communities of tomato plants (soil, *F* = 22.29, *R*^2^ = 0.7399, *p* < 0.001; cultivar, *F* = 2.04, *R*^2^ = 0.3223, *p* = 0.008). Of these two factors, soil explained a larger proportion of the compositional variance in the tomato rhizosphere microbiota. The results demonstrated that the assembly process of rhizosphere bacterial communities was collectively influenced by soil, including the available bacterial sources and biochemical properties of the rhizosphere soils, and plant genotype.

## 1. Introduction

As a plant habitat, soils are recognized as having the most diverse and abundant microbiota on the Earth [1]. Within soil–plant–root continuum ecosystems, three compartments can be distinguished: bulk soil, rhizosphere, and endosphere. These compartments generally contain rich sets of microbial species known as soil, rhizospheric, or endophytic microorganisms, respectively, with their definition based primarily on their relationship with the plant host [2,3,4,5]. Among these compartments, the rhizosphere has been most frequently studied because it represents the interface between the soil and plant roots. The rhizosphere microbiome plays a crucial role in bridging the soil and plant endophytic microbiomes [1,6,7,8].

Understanding the community composition and species diversity of rhizosphere microbiomes associated with different plant species is fundamental for maintaining a healthy rhizosphere environment and thus improving plant health and productivity [1,6,7,8]. Recently, the composition, assembly, variation and activities of the rhizosphere microbiome, as well as its interaction with host plant, have been studied in model plants and some crop species, such as *Arabidopsis* [2,3,9], rice [5,10], maize [11], potato [12,13,14], tomato [15] and soybean [16,17]. Based on surveys in a broad range of plant hosts, it has been widely accepted that the bacterial community and structure of the rhizosphere microbiome mostly remain stable between plants [1,5,18,19,20]. A large-scale replicated field study on the maize rhizosphere microbiome indicates that some of the core operational taxonomic units (OTUs) present in all the samples exhibited reproducible associations with plant genotype, regardless of the influences of field, plant age, and weather [20]. A comprehensive 16S rRNA gene-based amplicon sequencing survey has also suggested that some microbial taxa consistently associate with the rhizosphere microbiota of sorghum and sunflower grown in crop rotation in four different soils under field conditions [21].

The rhizosphere microbiota of the plant is mainly dominated by the bacterial phyla Proteobacteria, Bacteroidetes, and Acidobacteria [2,5,19,20]. In tomato, some studies on the community composition of the rhizosphere microbiota have reached the same conclusion using both culture-dependent and culture-independent approaches [15,22,23,24,25,26]. Qiao et al. [26] found that the three most abundant core phyla in the tomato (*Solanum lycopersicum* cv. Moneymaker) rhizosphere were Proteobacteria, Bacteroidetes, and Actinobacteria. More detailed information on the bacterial community composition and species diversity of the tomato rhizosphere microbiota was obtained for one tomato cultivar (*S. lycopersicum* cv. Jiabao) by using high-throughput sequencing. The results demonstrated that the tomato rhizosphere soil was dominated by the bacterial orders Sphingomonadales, Rhizobiales, Xanthomonadales, Burkholderiales, Cytophagales, and Sphingobacteriales in the phyla Proteobacteria, Bacteroidetes and Acidobacteria [15]. In a comprehensive study of 4 tomato cultivars (*S. lycopersicum*) from 4 geographically separated greenhouses, Lee et al. [25] compared rhizobacterial communities by using culture-dependent and culture-independent approaches, revealing that only 22% of the total OTUs in the MiSeq dataset were recovered in the culture collection and the most dominant phylum in the tomato rhizosphere microbiota was Proteobacteria (78.5%), followed by Actinobacteria (8.5%), Bacteroidetes (3.6%), and Acidobacteria (3.5%). Furthermore, Lee et al. [24] examined the microbial communities of three compartments of tomato plants collected from a broad range of geographical distributions. The results showed that the rhizosphere microbiota of the tomato plant was constituted by the bacterial phyla of Proteobacteria, Actinobacteria, Bacteoidetes, Fimicutes, and Acidobacteria.

The assembly and composition of rhizosphere microbial communities in tomato are affected by soil, plant genotype, and root system architecture [24,25,27,28,29]. In addition, the effects of pathogens, biocontrol microorganisms, and nutrient amendment on the tomato microbiota have been demonstrated [15,22,23]. Changes in the soil, such as land-use changes, will affect the assembly and final composition of rhizosphere microbial communities [27,28]. Allard et al. [22] and Cai et al. [23] suggested a correlation between the amended nutrient content in soils and changes in bacterial community structure in the rhizosphere of tomato plants. However, most studies on the tomato microbiome have focused on a few plant genotypes and experimental sites. A comprehensive and systematic evaluation of the community structure and composition, and the variation of the tomato rhizosphere microbiome across diverse host genotypes and soil environments, especially soils with extreme differences in nutrient amendment, has not been performed.

Regarding structure ecological communities in ecological research, several hypotheses, including the niche-based theory and neutral theory, have been proposed and discussed [30,31]. Recently, the niche-based mechanism has been observed in studies on the community composition and diversity of the microbiomes associated with the different niches plants [2,3,5,6,31,32,33,34]. By comparing the taxonomic and functional profiles of the microbiome associated with a particular niche, recent work revealed that the assembly of bacterial communities is a function-determined process. Bacterial community assembly in a specific niche is characterized by a consistent core functional profile, with features related to host-associated lifestyle. The assembly of the bacterial community is based on functional traits selected in the niche rather than a taxon [17,31,35,36]. In a particular niche, phylogenetically related or unrelated bacterial species with equivalent functional traits, as determined by the niche, are stochastically selected during the assembly of the bacterial community to determine the composition of the microbiome [17,31,35,36]. Both niche and neutral processes are likely to affect the assembly of complex microbial communities [30,34,35]. However, our understanding of the mechanism that shapes the tomato rhizosphere microbiome is still preliminary, and many fundamental questions remain unanswered. One such question is the degree to which plants and soil affect rhizosphere microbial communities.

In this study, we systematically evaluated the variation, community structures, and composition of the rhizosphere microbiotas associated with 11 tomato cultivars, and one cultivar grown in seven different soils. Revealing the detailed mechanisms that drive the assembly and composition of the rhizosphere microbiota will help us understand how the rhizosphere microbiomes in crop plants are modulated, which will guide the use of plant cultivar selection, land-use manipulation, soil management, universal biological control agents and fertilization for the development of more sustainable agricultural systems.

## 2. Materials and Methods

### 2.1. Plant Cultivars, Soils, and Plant Growth

In the cultivar experiment, 11 tomato cultivars were used, including 4 cultivars of tomato (*S. lycopersicum*), namely, Xinzhongshu No. 4 (XZ), Huangshoutao (HT), Tiancheng (TC) and Meiguodahong 168 (MG); and 7 cultivars of cherry tomato (*S. lycopersicum* var. cerasiforme), namely, Huapiqiu (HP), Huangshengnvguo (HS), Huangzhenzhu (HZ), Qiaokeli (QK), Yingtao (YT), Ziwucai (ZW), and Ziyixiannv (ZY) (Table S1). The tomato cultivars were grown in 3-liter pots (5-7 seeds per pot) filled with soil (Lawn soil, JX) collected from the Qishan campus of Fujian Normal University in Fuzhou, China. Seeds were surface sterilized with NaOCl (5%) for 10 min and thoroughly rinsed with sterile water before being planted. In the soil experiment, tomato cultivar MG was selected and grown in 7 different soils (or growing substrates), including natural field soils collected from five variable sites (campus lawn, garden, forest, agricultural field and vegetable field) around the Qishan campus of Fujian Normal University in Fuzhou, China, and two artificial commercial organic nutrient soils (general-purpose, peat and coconut bran based; Scotts Miracle-Gro, Marysville, OH, USA) as growing substrates (Table S2). The mineral composition of the soil samples was measured with an inductively coupled plasma atomic emission spectrometer as described by Pfeiffer et al. (Thermo Scientific, Waltham, MA, USA), and the organic matter, pH and available P content were determined as described by Cai et al. and Shen et al. (Table S3) [11,23,37].

In both experiments, the plants were grown in pots under natural light conditions. Fifty days after seed cultivation, the plants were pulled from pots, and the roots were shaken to remove large soil particles. The soil that tightly attached to the lateral root was scratched and carefully collected with a sterile filter paper stripe and used as the source of rhizosphere soil [38]. For each treatment, three replicates were collected. Therefore, a total of 54 rhizosphere soil (growing substrate) samples, 33 in the plant cultivar experiment and 21 in the soil experiment, were obtained for the subsequent extraction of total genomic DNA (Tables S1 and S2).

### 2.2. Metagenomic DNA Extraction

Metagenomic DNA was extracted from each of the rhizosphere soil samples using a Power Soil^®^ DNA Isolation Kit (MoBio Laboratories, Carlsbad, CA, USA) according to the manufacturer’s instructions. To assess the DNA quantity and purity, the DNA was run on 1% agarose gels at 90 V for 45 min in 1× TAE buffer; in addition, the concentration and purity of the DNA were measured using a NanoDrop 2000 spectrophotometer (Thermo Scientific). The samples of extracted total genomic DNA were stored at −20 °C until subjected to used high-throughput sequencing.

### 2.3. PCR Amplification and MiSeq High-Throughput Sequencing

DNA fragments of the V3-V4 region of the bacterial 16S rRNA gene were amplified using the primer pair 341F (5′-CCTACGGGNGGCWGCAG-3′) and 805R (5′-GACTACHVGGGTATCTAATCC-3′) fused with Illumina MiSeq adapters and a 6-bp barcode sequence unique to each sample [15,39]. PCR amplifications were carried out in triplicate in 50-μL reactions containing 10 ng of genomic DNA. The PCR amplification products were subsequently purified, combined in equimolar ratios, and subjected to high-throughput sequencing with the Illumina MiSeq sequencing platform, which produced paired-end 250-nucleotide reads, at Sangon Biotech (Shanghai, China).

### 2.4. Data Processing and 16S rRNA Gene-Based Community Analysis

Raw paired reads generated from a single library were initially merged using FLASH (version 1.2.3) [40], and then adaptors, barcodes, and primers were removed using Cutadapt (version 1.9.1). Low-quality reads (sequences with ambiguous bases, primer mismatches, average quality score < 25, or lengths shorter than 200 nt) were further removed using USEARCH (version 8.1.1861) [41]. After quality filtering, chimeric sequences were identified and removed by a de novo method using USEARCH (version 8.1.1861). The resulting data used for the processing steps are summarized in Tables S1 and S2.

To correct for differences in sequencing depth, bacterial read numbers per sample were separately rarefied on the basis of the sample with the smallest number of reads for subsequent analysis; 12,728 and 14,920 bacterial sequences were obtained for the cultivar and soil experiments, respectively. After subsampling, the data were processed according to a modified SOP pipeline based on USEARCH and QIIME [5,42]. Briefly, the selected sequences were clustered into OTUs using USEARCH (version 8.1.1861) at 97% sequence identity [41]. Representative sequences of each OTU were aligned to the SILVA reference database (release 128, updated September 2016) [43]. Taxonomy was subsequently assigned to each representative sequence using RDP classifier with a confidence greater than 90 [44]. 

The most dominant taxonomic unit and their relative abundances in each sample were visualized by drawing heatmaps based on the number of reads (log-transformed) using the R package “gplots” (https://cran.r-project.org/web/packages/gplots/index.html). The diversity and species richness indexes (alpha-diversity) for each sample, including OTU richness, the Chao1 index, the abundance-based coverage estimator (ACE), the Shannon index, and the Simpson index, were calculated using a QIIME script (Tables S4 and S5) [42]. Rarefaction and rank-abundance curves were generated at a 97% OTU similarity level. Statistical analysis was performed using analysis of variance (ANOVA) with *p* values to determine whether the diversity indexes were statistically significant among the rhizosphere soil samples [39]. To estimate beta diversity, the similarities of the members and structures of the microbiotas were calculated by utilizing the weighted and unweighted UniFrac distances from the normalized OTU tables for the various samples [42]. Unconstrained ordination analyses (principal coordinate analysis (PCoA) or principal component analysis (PCA)) were used to visualize the differences in bacterial community composition among the samples with a QIIME script or the R package ggbiplot (https://github.com/vqv/ggbiplot). The similarities of the soil samples based on their community members and structure were also found by using UniFrac-based hierarchical cluster analysis. The size and significance of the effects of soil and tomato cultivar on beta diversity were statistically measured using permutational MANOVA (PERMANOVA, adonis) or analysis of similarities (ANOSIM) with the QIIME pipeline [5]. Constrained analysis of principal components (CAP) was performed using the function “capscale” in the R package vegan [5]. Variance partitioning and significance testing were performed to determine the importance of soil and tomato cultivar for bacterial communities by running a PERMANOVA with 999 permutations. Furthermore, canonical correlation analysis (CCA) was used to determine which biochemical properties of the soils were significantly correlated with changes in microbial composition [45]. 

### 2.5. Nucleotide Sequence Accession Numbers

The datasets supporting the conclusions of this article are available in the National Center for Biotechnology Information (NCBI) repository (https://www.ncbi.nlm.nih.gov/bioproject/PRJNA316593/), under accession numbers SRR6214545 and SRR6214546. Data can be obtained from the BioSample database (https://www.ncbi.nlm.nih.gov/biosample), under accession numbers SAMN07832551 and SAMN07832551.

## 3. Results

### 3.1. Rhizosphere Communities Are Diverse and Consistent among Tomato Cultivars

**Figure 1:**
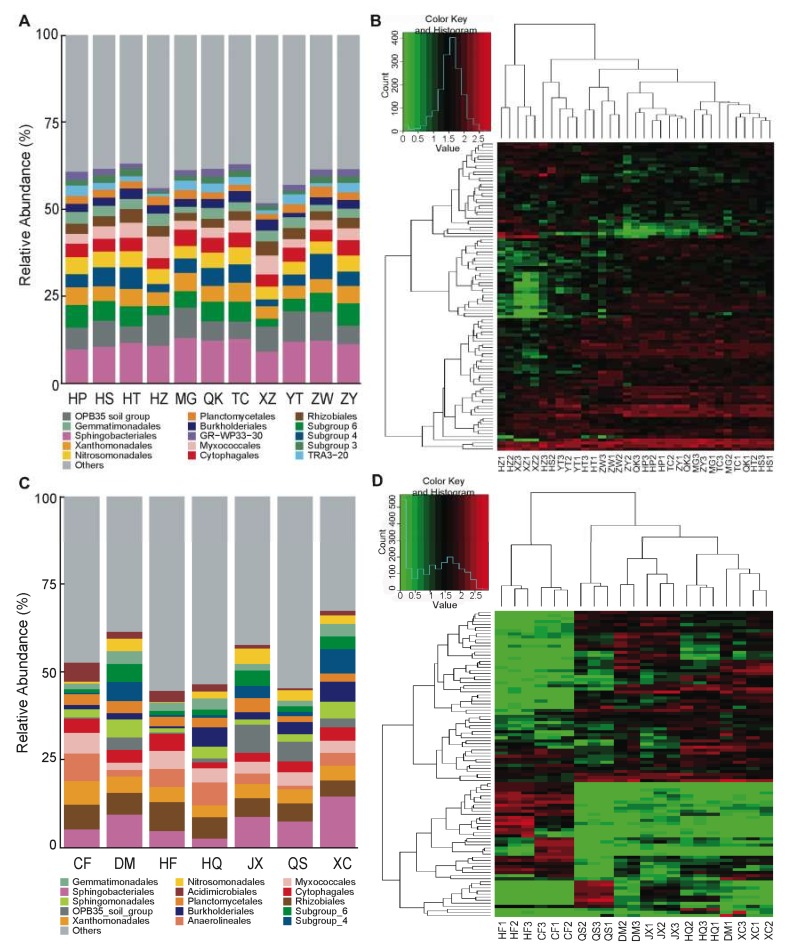
The composition and relative abundance of the major bacterial taxa and the 100 most abundant operational taxonomic units (OTUs) in the tomato rhizosphere microbiota. (**A**,**C**) The composition and relative abundance of major bacterial orders in tomato cultivar samples (**A**) or soil samples (**C**). Each bar represents the average value of three replicates in each sample group. (**B**,**D**) Heatmap depicting the 100 most abundant OTUs in the microbiota of tomato cultivar samples (**B**) or soil samples (**D**). Dendrogram links and distances between OTUs do not depict phylogenetic relationships; they are based on the number of reads (log-transformed) of OTUs within the samples. The legend and scale in the upper right corner of the figure show the colors in the heatmap associated with the relative abundance of OTUs (cluster of variables on the *Y*-axis) within each plant and soil sample (*X*-axis clustering). Tomato cultivar experiment: Xinzhongshu No. 4 (XZ), Huangshoutao (HT), Tiancheng (TC), Meiguodahong 168 (MG), Huapiqiu (HP), Huangshengnvguo (HS), Huangzhenzhu (HZ), Qiaokeli (QK), Yingtao (YT), Ziwucai (ZW) and Ziyixiannv (ZY); soil experiment: commodity organic nutritional soil (CF), potted plant nutrient soil (HF), vegetable field soil (DM), agricultural field soil (HQ), campus lawn soil (JX), forest soil (QS) and garden soil (XC).

The tomato rhizosphere microbiota was dominated by the bacterial phyla Proteobacteria (34.15 ± 2.68%), Bacteroidetes (16.41 ± 1.66%), and Acidobacteria (15.23 ± 3.66%), followed by Verrucomicrobia, Planctomycetes, Actinobacteria and Gemmatimonadetes (Figure S1A). Proteobacteria was the predominant phylum in the tomato rhizobacterial communities with abundant Xanthomonadales (4.52 ± 0.64%), Nitrosomonadales (4.26 ± 0.54%), Myxococcales (3.63 ± 1.23%), Rhizobiales (3.01 ± 0.57%), and Burkholderiales (2.46 ± 0.56%). Sphingobacteriales (11.48 ± 1.23%) and Cytophagales (3.90 ± 0.50%) in Bacteroidetes and subgroup 6 (4.94 ± 1.47%) and subgroup 4 (4.44 ± 1.62%) in Acidobacteria were the dominant groups (Figure 1A and Figure S1B–E). The tomato cultivar rhizosphere microbiotas showed similar compositions and structures.

The core members of the rhizosphere microbiotas within the tomato cultivar samples were investigated. The number of identified core OTUs decreased with an increasing sample number (Figure S2A). Accordingly, core OTUs were defined as OTUs that were present in at least 85% of the samples. A total of 655 OTUs belonging to 68 bacterial orders of 14 phyla were identified. These identified core OTUs were primarily from the bacterial orders Sphingobacteriales, subgroups 4 and 6, Xanthomonadales, Nitrospirales, Cytophagales, Rhizobiales, Burkholderiales and TRA3-20 from the phyla Proteobacteria, Bacteroidetes and Acidobacteria (Table S6). Core microbiome analysis indicated that the defined core OTUs in all 11 tomato cultivars accounted for 72.50% of the total rarefied reads in the cultivar experiment. Among the OTUs, 291 could be identified in all samples.

The consistent community composition of the rhizosphere microbiotas among cultivar samples was observed not only for the core OTUs but also for the dominant OTUs. A heatmap was constructed to illustrate the relative abundance of the 100 most abundant OTUs, which accounted for 38.64% of the total rarefied reads in the cultivar experiment (Figure 1B). The most abundant OTUs also belonged to the identified core groups: Sphingomonadales and Cytophagales of Bacteroidetes; Xanthomonadales and Nitrosomonadales of Proteobacteria; and subgroup 4 and subgroup 6 of Acidobacteria (Figure 1B and Table S7). The relative abundances of the most abundant OTUs were generally consistent among cultivar rhizosphere microbiotas. A few OTUs showed a differential abundance among samples; for example, some OTUs were unique to cultivar XZ, exhibiting a low relative abundance (Figure 1B and Table S7). Cluster analysis based on the most abundant OTUs did not reveal any tendencies for different samples to be grouped according to tomato varieties or cultivars (Figure 1B).

Bacterial diversity in the samples (alpha diversity), as measured by the Chao1 index, Shannon index, Simpson’s index and ACE, was evaluated using OTU-based analysis (Figure S3A and Table S4). The rarefaction curves showed that the samples reached the saturation phase, with a satisfactory level of confidence and that most OTUs in each sample were detected (Figure S4A). Furthermore, Good’s coverage was above 92%, indicating an adequate sequencing depth (Table S4). The bacterial diversity of the rhizosphere microbiota, which was estimated by the Shannon index (*p* = 0.19), varied slightly among tomato cultivars, but no statistically significant differences were observed. Only some of the cultivars, such as HS, HT, YT, and ZW, exhibited a statistically significant difference compared with cultivars HP, QK, and ZY (*p* < 0.05). However, the OTU richness estimated with the Chao1 index (*p* = 0.0043 < 0.05) differed significantly among the tomato cultivars. Rank-abundance curves were constructed to visually depict both species richness and evenness in different tomato cultivar rhizosphere microbiotas (Figure S3B). Different tomato cultivar samples exhibited similar species richness and evenness values.

### 3.2. The Influence of Soil (Growing Substrate) on the Assembly and Composition of the Rhizosphere Microbiota in Tomato

The influence of soil on the assembly and composition of the rhizosphere microbiota was evaluated by growing *S. lycopersicum* cv. MG in soils from seven different sources. The tomato plants grown in different soils harbored rhizobacterial communities that varied greatly in structure and composition. As shown in the cultivar experiment, the tomato rhizosphere microbiota was dominated by bacteria from the phyla Proteobacteria (34.86 ± 4.48%), Bacteroidetes (13.37 ± 5.16%), Acidobacteria (12.69 ± 9.81%) and Actinobacteria (8.73 ± 5.09%), mainly comprising Rhizobiales, Xanthomonadales, Burkholderiales, Nitrosomonadales, Myxococcales, Sphingomonadales, Sphingobacteriales, Cytophagales and Acidobacteria subgroups (Figure 1C and Figure S5A–E). The proportions of dominant bacterial communities in the soils differed greatly at both the phylum and order levels (Figure 1C and Figure S5A–E). However, the assembly of the cultivar rhizosphere microbiotas in the cultivar experiment mostly overlapped with that in tomato cultivar MG grown in the five natural field soils DM, JX, HQ, QS and XC (Figure 1A,C and Figures S1B–E and S5B–E). The commercial nutrient soils HF and CF harbored distinct bacterial communities and rhizosphere microbiota compositions, with the dominant bacterial groups being Acidimicrobiales of Actinobacteria and Caulobacterales, Rhizobiales, Myxococcales, and Xanthomonadales of Proteobacteria (Figure 1C,D). The bacterial communities varied significantly between the five natural field soils and two artificial commercial nutrient soils in the dominant bacterial groups of Myxococcales, Burkholderiales and Nitrosomonadales of Proteobacteria, subgroup 6 of Acidobacteria, Acidimicrobiales of Actinobacteria, and Flavobacteriales of Bacteroidetes (*p* < 0.05).

The alpha diversities of the tomato rhizosphere microbiotas for all seven soil samples were measured (Figure S3C and Table S5). The rarefaction curves showed that all of the samples reached the saturation phase with a satisfactory level of confidence and Good’s coverage of at least 95% (Figure S4b). Significant differences in OTU richness estimated by the Chao1 index (*p* < 0.0001) and species diversity determined by the Shannon index (*p* = 0.0014 < 0.01) were observed among the seven soil samples (Figure S3C and Table S5). However, no significant differences in species diversity (Shannon index: *p* = 0.19) were observed among the five natural soils DM, HQ, JX, XC, and QS. Rank-abundance curves were constructed to visually depict both species richness and evenness in the seven different soil samples (Figure S3D). Soil samples DM, HQ, JX, and XC exhibited higher species richness and species evenness than the HF and CF samples, suggesting that the bacterial species in the DM, HQ, JX and XC soils were more diverse and evenly distributed than those in the HF and CF soils.

### 3.3. Variation in Core Communities and Dominant OTUs in the Rhizosphere Microbiota among Soil Environments

Analysis of the core member of the rhizosphere microbiota of the same tomato cultivar MG grown in different soils at the 85% confidence interval level revealed a total of 105 OTUs; these OTUs belonged to 27 orders of 9 phyla and accounted for only 18.53% of the total rarefied reads in the soil experiment (Table S6 and Figure S2B,C). Some of the core bacteria identified in the cultivar experiment, such as Cytophagales, Gemmatimonadetes, Anaerolineales and subgroup 3, were absent in the soil experiment. However, if the commercial soil samples HF and CF were excluded, more than 333 core OTUs, representing 45.83% of the total rarefied reads and belonging to 51 orders of 13 bacterial phyla, could be found among the five natural field soil samples (DM, JX, HQ, QS and XC) (Table S6). Few core OTUs were shared between the commercial soil samples and natural field soil samples (Figure S2B,C).

The heatmap using the relative abundance of the 100 most abundant OTUs, which accounted for 29.8% of the total rarefied reads in the soil experiment, revealed high variation in the relative abundance of OTUs among the soil samples (Figure 1D). Cluster analysis based on the most abundant OTUs revealed that the samples were significantly clustered according to soil sources (Figure 1D). The samples were separated into two groups based on the composition and relative abundance of OTUs. One group included the two commercial nutritional soils HF and CF. DM, HQ, JX, QS and XC, the natural soil samples constituted another group, which was further separated into two subsets QS (forest soil) and DM, HQ, JX and XC (field soils, including agricultural and vegetable field, garden and lawn soils).

The core OTUs and dominant OTUs in each of the communities differed greatly between the natural field soils and commercial nutrient soils. The commercial nutrient soils HF and CF contained fewer of the OTUs belonging to the Nitrosomonadales, Burkholderiales, and Rhodospirillales of Proteobacteria and Acidobacteriales and other subgroups of Acidobacteria but were significantly enriched in OTUs belonging to Myxococcales, Methylophilales, and Oceanospirillales of Proteobacteria, Acidimicrobiales of Actinobacteria, and Cytophagales of Bacteroidetes compared with the field soil samples (Figure 1D and Table S8). Moreover, the dominant OTUs in the rhizobacterial communities in the two categories of soil samples also varied. For example, the most abundant OTUs in Sphingomonadales, Rhizobiales, Burkholderiales, and Actinobacteria in the rhizosphere microbiota were OTU17 (*Sphingomonas*), OTU54 (*Hyphomicrobium*), OTU53 (*Limnobacter*), and OTU6 (*Actinomadura*) in the commercial nutritional soils but OTU15 (*Sphingomonas*), OTU7 (*Bradyrhizobium*), OTU25 (*Massilia*), and OTU2 (*Arthrobacter*) in the natural field soils (Figure 1D and Table S8). The core and dominant OTUs were always shared by the same kinds of soil (natural field soils or commercial nutrient soils). Even for the core OTUs identified in both categories of soil samples, different dominant OTUs belonging to the same bacterial taxon were enriched in the other category of soil samples.

### 3.4. Evaluating the Effects of Tomato Cultivar or Soils on Tomato Rhizosphere Microbiota Based on the Combined Data

**Figure 2:**
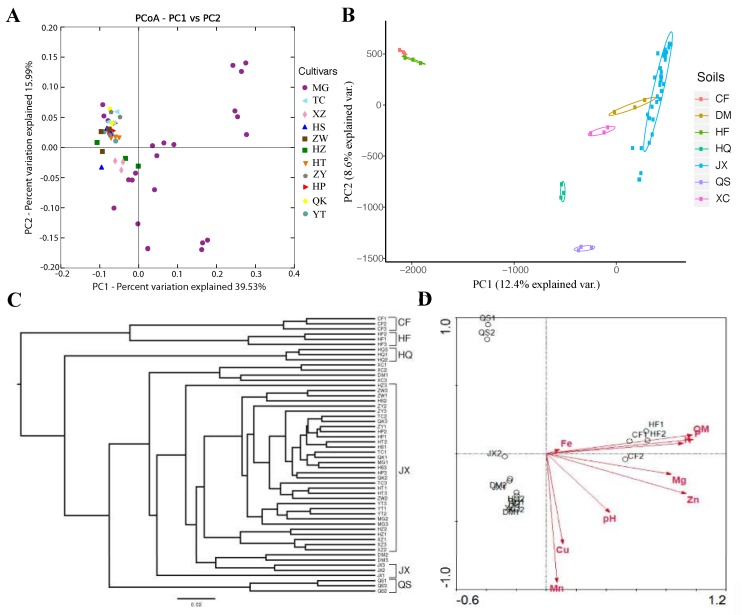
Beta diversity analysis to estimate the dissimilarity and similarity of bacterial community compositions among the cultivars and soil samples. (**A**) Principal coordinate analysis (PCoA) derived from the dissimilarity matrix of weighted UniFrac distances based on the combined data of the cultivars and soil samples. (**B**) Principal component analysis (PCA) showing sample grouping based on soil source from the combined data of the cultivars and soil samples. (**C**) Weighted UniFrac-based hierarchical cluster analysis of bacterial community composition based on the combined data of the cultivars and soil samples. (**D**) Canonical correspondence analysis (CCA) showing the main soil biochemical factors that affected the assembly and composition of the rhizobacterial communities based on the combined data of the cultivars and soil samples. Tomato cultivar experiment: Xinzhongshu No. 4 (XZ), Huangshoutao (HT), Tiancheng (TC), Meiguodahong 168 (MG), Huapiqiu (HP), Huangshengnvguo (HS), Huangzhenzhu (HZ), Qiaokeli (QK), Yingtao (YT), Ziwucai (ZW) and Ziyixiannv (ZY); soil experiment: commodity organic nutritional soil (CF), potted plant nutrient soil (HF), vegetable field soil (DM), agricultural field soil (HQ), campus lawn soil (JX), forest soil (QS) and garden soil (XC).

To show the degree to which the plant genotypes and soils impacted the rhizosphere bacterial communities of tomato roots, the data from both the tomato cultivar and soil experiments, including those for all the cultivars and soil samples, were combined. Alpha-diversity analysis based on the combined data demonstrated that the rhizobacterial communities of tomato grown in the same soil showed similar species richnesses. In contrast, the rhizobacterial communities of the same tomato cultivar grown in different soils showed greater variation in species richness (Figure S4C,D). Unconstrained ordination analyses (PCoA or PCA) revealed the similarities of the rhizosphere microbial structure and composition among the tomato cultivars or soil samples (Figure 2A,B and Figure S6A,B). A tendency for the samples to be grouped was found with PCoA in the soil experiment (Figure S6B). PCoA and PCA based on the combined data from the cultivar and soil experiments also grouped the samples based on the soil source but not based on genotype (Figure 2A,B). Similar results were also found with hierarchical cluster analysis, which was performed using QIIME (Figure 2C and Figure S6C,D). The samples were clustered into different groups to a greater extent by soil source more than by plant cultivar. Soil samples CF and HF formed an independent branch, and QS further formed a single group. The other soil samples, HQ, DM, JX, and XC, including all the tomato cultivar samples grown in soil JX, clustered together (Figure 2C and Figure S6C,D).

The results of statistical analysis of beta diversity based on the combined data were in agreement with those from the ordination analysis and the hierarchical cluster analysis. Soil had a significant impact on the rhizosphere microbial communities of tomato plants based on weighted UniFrac distances (ANOSIM, *R* = 0.9584, *p* < 0.001; Adonis, *F* = 22.29, *R*^2^ = 0.7399, *p* < 0.001) and unweighted UniFrac distances (ANOSIM, *R* = 0.9303, *p* < 0.001; Adonis, *F* = 8.15, *R*^2^ = 0.5098, *p* < 0.001). Plant genotype (cultivar) had a significant impact on the rhizosphere microbial communities with little explanation of the difference among the samples in the Adonis analysis (weighted, *F* = 2.04, *R*^2^ = 0.3223, *p* = 0.008; unweighted, *F* = 1.80, *R*^2^ = 0.2956, *p* < 0.001). No statistically significant differences were found among cultivar samples with ANOSIM (weighted, *p* = 1.00; unweighted, *p* = 0.99). Results of CAP constrained to cultivars and soils agreed with the PERMANOVA (Adonis) results that there were significant differences in microbial communities among soil and cultivar samples (soil: 26.55% of the variance, *p* = 0.002; cultivar: 15.77% of the variance, *p* = 0.002).

To further investigate the soil properties that affected the rhizosphere microbiota, the biochemical properties of the soil samples were determined (Table S3). The two commercial nutrient soils (CF and HF), with higher organic matter, P and K contents than the other soils, showed similar biochemical properties. While four of the other sources of soil (DM, HQ, JX, and XC) had similar soil chemical contents and properties, the forest soil (QS) had the lowest Cu, Mn, P, Zn, and Mg contents. These patterns of chemical contents and properties resulted in two categories of soil samples, commercial nutrient soils (CF and HF) and natural field soils (DM, HQ, JX, XC, and QS). The soil categories corresponded to the groups based on the divergence in taxonomic composition and abundance. CCA revealed that among the examined soil chemical properties, organic matter, P and K were the main soil environmental factors that affected the assembly and composition of the rhizobacterial communities in the different samples and the main determinants separating the commercial soil samples CF and HF from the others soil samples (*p* < 0.01) (Figure 2D). The differential organic matter, P, K and Mn contents in the different rhizosphere soil samples clearly explained the distinct microbial community compositions, species richnesses and bacterial diversities in the commercial soils CF and HF compared to those in the natural field soil samples DM, HQ, JX, and XC and the forest soil sample QS in the previous analysis.

## 4. Discussion

It is widely accepted core microbial communities presented in the gut microbiomes of humans and other animals, as well as root microbiomes of plants [4,5,7,8,19,20]. Assembly of rhizosphere microbiome in plant is driven by many aspects, including climate environment, soil source, host developmental stage, cultivation practice, and root architecture [5,6,7,8,11,14,21,22,23,24]. Biotic factors, such as plant genotypes, pathogens, biocontrol microorganisms, and seed bacteria also alter and influence the microbial communities in rhizosphere environment [5,6,7,8,12,13,14,26,27,28,29,30,46,47,48]. Previous studies have shown that plant and soil are both important factors in shaping the community structure of rhizosphere microbiota [14]. In this study, we investigated the effects of a broad range of cultivars and soils (growing substrates) on microbial communities of the rhizosphere microbiota in tomato. We minimized biases and effects from technical variation, sampling, and climate environment by processing seeds with surface sterilization, using the standardized sample collection protocol, and growing the tomato plants in pots rather than fields with routine management of watering and light under a greenhouse.

Similar community compositions of rhizosphere microbiotas demonstrated in tomato cultivars. The tomato rhizosphere microbiota mainly composed Proteobacteria, Bacteroidetes, and Acidobacteria. Moreover, the identified core OTUs mostly belonged to the most abundant groups, including Acidobacteria subgroups 4 and 6, Sphingobacteriales, Xanthomonadales, Nitrospirales, Cytophagales, Rhizobiales and Burkholderiales from the dominant bacterial phyla. The results showed that the rhizobacterial communities of different plant cultivars mainly differed in the abundances of the bacteria, not their community assembly. Statistical analysis exhibited a significant difference in the OTU richness (*p* < 0.05), rather than bacterial diversity, among the rhizosphere microbiotas of tomato cultivars. The bacterial communities in the five natural field soils DM, JX, HQ, QS and XC, but not the two commercial soils, mostly overlapped with those of the rhizosphere microbiotas in the different cultivar samples. These results were consistent with previous observations of the rhizosphere microbiota of other tomato cultivars, which were dominated by the bacterial orders Sphingomonadales, Rhizobiales, Xanthomonadales, Burkholderiales, Cytophagales and Sphingobacteriales from the bacterial phyla Proteobacteria, Bacteroidetes, and Acidobacteria [15,25]. The result supported the hypothesis that tomato harbors largely conserved communities of rhizosphere microbes that remain stable among cultivars of tomato and even among the field soils from different sources [24,25,26]. However, compared to the natural soil samples, the commercial nutrient soil samples HF and CF harbored distinct bacterial communities and compositions, with significant variation in Myxococcales, Burkholderiales and Nitrosomonadales of Proteobacteria, subgroup 6 of Acidobacteria, Acidimicrobiales of Actinobacteria, and Flavobacteriales of Bacteroidetes. Statistical analysis demonstrated that soil had a significant effect on both the OTU richness and bacterial diversity of the tomato rhizosphere microbiota. 

Previous studies have demonstrated the prevalent role of soil in shaping the assembly and composition of the rhizosphere microbiome [49,50,51,52]. In this study, PERMANOVA for beta diversity based on the combined data from the cultivar and soil experiments demonstrated that soil and plant genotype (cultivar) had a significant impact on the rhizosphere microbial communities of tomato plants. However, hierarchical cluster analysis and PCA clustered the samples into different groups based on soils rather than plant genotype. Results of CAP and PCoA further indicated that soil factors explained a large proportion of the variance in the composition of the tomato rhizosphere microbiota. In rhizosphere, soil provides bacterial sources for plant selectively modulating its microbial communities [2,3,5,11]. Plant recruits and enriches the rhizobacterial species by releasing root exudates into the soils surrounding roots [7]. It has been shown that the phytochemical content of root exudates is related to the plant phylogeny [53]. This explained that cultivars of tomato plant harbored a similar community composition. The relative abundance of core OTUs and rhizobacterial communities in different natural soils and under different cultivars may reflect changes of phytochemicals content releasing by roots. Furthermore, changes in the soil and the amended nutrient content in soils will result in changes of bacterial inocula presented in soils, and correspondingly changes happen in the community structure of the rhizosphere in tomato plants [22,23,28]. The artificial nutrient soils HF and CF showed distinct organic matter, P, K, and Mn contents compared with those of the five natural field soils. CCA showed that organic matter, P and K were the main soil environmental factors that affected the assembly and composition of the rhizobacterial communities in the different samples. The nutrient soils and the natural field soils harbored different microbial sources or systems, allowing the plants to selectively modulate their rhizosphere microbiome. Therefore, distinct dominant species or OTUs were enriched and recruited to assemble the bacterial communities in the rhizosphere microbiota of tomato grown in the HF and CF soils.

Core microbiome analysis showed a total of 655 OTUs accounted for 72.50% of the total rarefied reads in the cultivar experiment, and a total of 333 OTUs, 45.83% of the total rarified reads were shared among the five natural field soil samples. The similar bacterial communities and consistent dominant and core OTUs in the rhizosphere microbiotas of different cultivars or the cultivar grown in the five natural field soils demonstrated the tendency of shaping microbiome for cultivars of plant species to selectively enrich specific microbes. The results supported the niche-based hypothesis that plants have the ability to adapt to and selectively recruit and enrich rhizobacterial members in order to promote microbial activities that can enhance their fitness in variable soil environments [31,33,36]. However, further analysis of the bacterial communities of tomato plants grown in the artificial commercial nutrient soils HF and CF showed that the ability of the plants to selectively modulate the rhizosphere microbiota was limited by the available bacterial sources present in the soils. Plants enriched and recruited different dominant species or OTUs, forming distinct bacterial communities and compositions with different dominant OTUs when grown in the artificial commercial nutrient soils HF and CF, Plants showed the ability to adapt to the variable environment by recruiting other functionally equivalent taxonomic groups to the rhizosphere, not restricted to particular taxonomic groups [31,33]. Both niche and neutral processes are likely to be involved in the assembly process of bacterial communities in the rhizosphere. However, the selection of different bacterial members from the HF and CF soils was not random. The dominant OTUs in the HF and CF soils were not always the same as those in the natural field soils, even if one taxon was identified as a core OTU and shared by the two categories of soil samples. In most cases, the most abundant OTUs in the dominant bacterial groups were shared by members of one soil sample category, suggesting that the assembly process and compositions of bacterial communities were also influenced by the soil, including the available bacterial sources and biochemical traits of the rhizosphere soils and plant genotype. 

## Supplementary Materials

The following are available online at https://www.mdpi.com/2076-2607/8/2/170/s1, Table S1: Summary of cultivar samples and data statistics for the read processing steps in the tomato cultivar experiment, Table S2: Summary of soil samples and data statistics for the read processing steps in soil experiment, Table S3: Basic biochemical parameters of the soil samples, Table S4: Summary of species richness and diversity indexes for all the samples in cultivar experiment, Table S5: Summary of species richness and diversity indexes for all samples in soil experiment, Table S6: Taxonomic compositions and relative abundances of the core OTUs identified in the cultivar and soil experiments, respectively, Table S7: Taxonomic identification of the most abundant 100 OTUs in the microbiota of tomato cultivar samples in cultivar experiment, Table S8: Taxonomic identification of the most abundant 100 OTUs in the microbiota of soil samples in soil experiments, Figure S1: The composition and relative abundance of the major bacterial taxa in the tomato cultivar samples in cultivar experiment. Each bar represents the average value of three replicates in each sample group. (**A**) The composition and relative abundance of major bacterial phyla; (**B**–**D**) The composition and relative abundance of the major bacterial orders from the phyla of Bacteriodetes (**B**), Acidobacteria (**C**) and Proteobacteria (**D**); (**E**) The composition and relative abundance of the major bacterial orders from four classes of the phylum Proteobacteria: Alpha-, Beta-, Delta- and Gamma-proteobacteria. Xinzhongshu No. 4 (XZ), Huangshoutao (HT), Tiancheng (TC), Meiguodahong 168 (MG), Huapiqiu (HP), Huangshengnvguo (HS), Huangzhenzhu (HZ), Qiaokeli (QK), Yingtao (YT), Ziwucai (ZW) and Ziyixiannv (ZY), Figure S2: The percentage of core OTUs to be presented in the samples. (**A**) Tomato cultivar experiment; (**B**) Soil experiment; (**C**) soil experiment except for samples CF and HF. Figure S3. The OTU number boxplots and the rank abundance curves indicate OTU richness of the sample in the cultivar and soil experiments, respectively. The plots were drawn using the average value of three replicates of each sample group. (**A**) The OTU number boxplot depicting OTU richness in the microbiota of tomato cultivar samples; (**B**) The rank-abundance curve depicting species richness and evenness in the microbiota of tomato cultivar samples; (**C**) The OTU number boxplot depicting OTU richness in the microbiota of soil samples; (**D**) The rank-abundance curve depicting species richness and evenness in the microbiota of soil samples. Tomato cultivar experiment: Xinzhongshu No. 4 (XZ), Huangshoutao (HT), Tiancheng (TC), Meiguodahong 168 (MG), Huapiqiu (HP), Huangshengnvguo (HS), Huangzhenzhu (HZ), Qiaokeli (QK), Yingtao (YT), Ziwucai (ZW) and Ziyixiannv (ZY); soil experiment: commodity organic nutritional soil (CF), potted plant nutrients soil (HF), vegetable field soil (DM), agricultural field soil (HQ), campus lawn (JX), forest soil (QS), garden soil (XC), Figure S4: Rarefaction curves and the OTU number boxplot of the rhizosphere microbiota in the cultivar or soil experiments. (**A**) Rarefaction curves of the different tomato cultivar samples in cultivar experiment; (**B**) Rarefaction curves of the different sources of soil samples in soil experiment; (**C**) Rarefaction curves of all samples in both the cultivar and soil experiments; (**D**) The OTU number boxplot of all samples in both the cultivar and soil experiments, Figure S5: The composition and relative abundance of the major bacterial taxa in the soil samples in soil experiment. Each bar represents the average value of three replicates in each sample group. (**A**) The composition and relative abundance of the major bacterial phyla; (**B**–D) The composition and relative abundance of the major bacterial orders from the phyla of Bacteriodetes (B), Acidobacteria (**C**) and Proteobacteria (**D**); (**E**) The composition and relative abundance of the major bacterial orders from four classes of the phylum Proteobacteria: Alpha-proteobacteria, Beta-proteobacteria, Delta-Proteobacteria and Gamma-proteobacteria. Commodity organic nutritional soil (CF), potted plant nutrients soil (HF), vegetable field soil (DM), agricultural field soil (HQ), campus lawn (JX), forest soil (QS) and garden soil (XC), Figure S6: Beta diversity analysis to estimate the dissimilarity and similarity of bacterial communities and composition among the samples, (**A**) Principal coordinated analysis (PCoA) derived from the dissimilarity matrix of the weighted UniFrac distance among the cultivar samples in cultivar experiment, (**B**) The hierarchical cluster analysis of the bacterial community composition among the cultivar samples in cultivar experiment, (**C**) Principal coordinated analysis (PCoA) derived from the dissimilarity matrix of the weighted UniFrac distance among the soil samples in soil experiment, (**D**) The hierarchical cluster analysis of the bacterial community composition among the soil samples in soil experiment.
