# Supplementary material for: Revealing the Variation and Stability of Bacterial Communities in Tomato Rhizosphere Microbiota
Source: Microorganisms. 2020 Jan 25;8(2):170. doi: 10.3390/microorganisms8020170 (PMC7074737; doi:10.3390/microorganisms8020170)

Supplementary Information

Table S1 Summary of cultivar samples and data statistics for the read processing steps in tomato cultivar experiment.

Sample ID	Tomato varieties	Tomato cultivar	Soil	Raw reads	Clean reads
XZ1	*Solanum lycopersicum*	Xinzhongshu No. 4	Lawn, Qishan (JX)	28411	18712
XZ2	*Solanum lycopersicum*	Xinzhongshu No. 4	Lawn, Qishan (JX)	24513	15471
XZ3	*Solanum lycopersicum*	Xinzhongshu No. 4	Lawn, Qishan (JX)	27909	17746
MG1	*Solanum lycopersicum*	Meiguodahong 168	Lawn, Qishan (JX)	25638	15540
MG2	*Solanum lycopersicum*	Meiguodahong 168	Lawn, Qishan (JX)	26639	17478
MG3	*Solanum lycopersicum*	Meiguodahong 168	Lawn, Qishan (JX)	22169	15690
HP1	*Solanum lycopersicum var. cerasiforme*	Huangpiqiu	Lawn, Qishan (JX)	28568	16739
HP2	*Solanum lycopersicum var. cerasiforme*	Huangpiqiu	Lawn, Qishan (JX)	26316	16833
HP3	*Solanum lycopersicum var. cerasiforme*	Huangpiqiu	Lawn, Qishan (JX)	27349	15996
HS1	*Solanum lycopersicum var. cerasiforme*	Huangshengnvguo	Lawn, Qishan (JX)	25517	14498
HS2	*Solanum lycopersicum var. cerasiforme*	Huangshengnvguo	Lawn, Qishan (JX)	20749	12728
HS3	*Solanum lycopersicum var. cerasiforme*	Huangshengnvguo	Lawn, Qishan (JX)	28267	16903
HT1	*Solanum lycopersicum*	Huangshoutao	Lawn, Qishan (JX)	26674	16069
HT2	*Solanum lycopersicum*	Huangshoutao	Lawn, Qishan (JX)	26768	16546
HT3	*Solanum lycopersicum*	Huangshoutao	Lawn, Qishan (JX)	27751	17200
HZ1	*Solanum lycopersicum var. cerasiforme*	Huangzhenzhu	Lawn, Qishan (JX)	24884	16568
HZ2	*Solanum lycopersicum var. cerasiforme*	Huangzhenzhu	Lawn, Qishan (JX)	26258	17633
HZ3	*Solanum lycopersicum var. cerasiforme*	Huangzhenzhu	Lawn, Qishan (JX)	24790	16288
QK1	*Solanum lycopersicum var. cerasiforme*	Qiaokeli	Lawn, Qishan (JX)	29394	18035
QK2	*Solanum lycopersicum var. cerasiforme*	Qiaokeli	Lawn, Qishan (JX)	28477	17485
QK3	*Solanum lycopersicum var. cerasiforme*	Qiaokeli	Lawn, Qishan (JX)	23552	14081
TC1	*Solanum lycopersicum*	Tiancheng	Lawn, Qishan (JX)	24843	14616
TC2	*Solanum lycopersicum*	Tiancheng	Lawn, Qishan (JX)	24441	15109
TC3	*Solanum lycopersicum*	Tiancheng	Lawn, Qishan (JX)	27164	16295
YT1	*Solanum lycopersicum var. cerasiforme*	Yingtao	Lawn, Qishan (JX)	28020	18014
YT2	*Solanum lycopersicum var. cerasiforme*	Yingtao	Lawn, Qishan (JX)	28162	18888
YT3	*Solanum lycopersicum var. cerasiforme*	Yingtao	Lawn, Qishan (JX)	28380	19424
ZW1	*Solanum lycopersicum var. cerasiforme*	Ziwucai	Lawn, Qishan (JX)	25268	16009
ZW2	*Solanum lycopersicum var. cerasiforme*	Ziwucai	Lawn, Qishan (JX)	26187	15442
ZW3	*Solanum lycopersicum var. cerasiforme*	Ziwucai	Lawn, Qishan (JX)	26141	16555
ZY1	*Solanum lycopersicum var. cerasiforme*	Ziyixiannv	Lawn, Qishan (JX)	26296	15282
ZY2	*Solanum lycopersicum var. cerasiforme*	Ziyixiannv	Lawn, Qishan (JX)	27305	16177
ZY3	*Solanum lycopersicum var. cerasiforme*	Ziyixiannv	Lawn, Qishan (JX)	29874	17870

Table S2 Summary of soil samples and data statistics for the read processing steps in soil experiment.

Sample ID	Tomato	Soil sample	Soil category	Raw reads	Clean reads
CF1	Meiguodahong 168 (MG)	Commodity organic nutritional soil	Commercial nutrients soil	30566	22487
CF2	Meiguodahong 168 (MG)		Commercial nutrients soil	30305	21730
CF3	Meiguodahong 168 (MG)		Commercial nutrients soil	30565	21885
DM1	Meiguodahong 168 (MG)	Vegetable field, East star village in FZ	Natural field soil	28269	16995
DM2	Meiguodahong 168 (MG)		Natural field soil	32127	19748
DM3	Meiguodahong 168 (MG)		Natural field soil	32009	19051
HF1	Meiguodahong 168 (MG)	Potted plant nutrients soil	Commercial nutrients soil	33852	22977
HF2	Meiguodahong 168 (MG)		Commercial nutrients soil	29436	19241
HF3	Meiguodahong 168 (MG)		Commercial nutrients soil	27949	20106
HQ1	Meiguodahong 168 (MG)	Agriculture field, Hongtang Bridge North in FZ	Natural field soil	27254	17633
HQ2	Meiguodahong 168 (MG)		Natural field soil	26366	17515
HQ3	Meiguodahong 168 (MG)		Natural field soil	26368	17355
JX1	Meiguodahong 168 (MG)	Lawn, Qishan Campus of FNU	Natural field soil	28769	18203
JX2	Meiguodahong 168 (MG)		Natural field soil	25124	14920
JX3	Meiguodahong 168 (MG)		Natural field soil	29707	19291
QS1	Meiguodahong 168 (MG)	Forest, Qishan forest park in Fuzhou	Natural field soil	32130	19751
QS2	Meiguodahong 168 (MG)		Natural field soil	28170	18167
QS3	Meiguodahong 168 (MG)		Natural field soil	42677	26567
XC1	Meiguodahong 168 (MG)	Garden soil in Minhou, FZ	Natural field soil	29513	19609
XC2	Meiguodahong 168 (MG)		Natural field soil	30159	19913
XC3	Meiguodahong 168 (MG)		Natural field soil	29906	19520

Table S3 Basic biochemical parameters of the soil samples.

Sample ID	Organic matter(%)	pH	P(mg/kg)	Zn(mg/kg)	Mn(mg/kg)	Cu(mg/kg)	Fe(mg/kg)	K(mg/kg)	Mg(mg/kg)
CF-1	31.93	6.74	933.09	183.61	620.28	31.87	45537.03	6025.04	40560.82
CF-2	30.79	6.8	942.91	176.82	592.74	31.87	38937.23	5032.41	34426.91
CF-3	30.82	6.67	928.15	183.28	509.23	32.63	39307.43	5723.84	40016.27
DM-1	2.29	6.78	106.08	140.32	665.06	31.92	28217.96	3060.03	18476.88
DM-2	2.13	6.83	106.57	130.8	625.75	28.24	27685.28	1869.38	18153.78
DM-3	1.89	6.65	107.91	136.52	655.19	29.52	27912.48	2252.36	22612.62
HF-1	27.89	7.05	1368.7	185.7	561.77	25.17	24138.34	10844.15	77439.58
HF-2	28.44	7.13	1368.69	205.15	609.74	26.5	25611.36	11782.79	85016.66
HF-3	28.12	7.12	1346.01	198.12	594.03	26	24321.26	9706.74	80572.16
HQ-1	0.9	6.67	19.64	127.01	839.39	24.52	36865.54	1285.54	21959.87
HQ-2	0.87	6.64	18.66	125.2	827.84	24.25	33999.07	1301.41	50945.2
HQ-3	0.93	6.38	20.23	122.22	843.56	23.85	35566.91	1104.78	69723.96
JX-1	2.42	7.07	35.80	121.4	782.36	22.63	31985	1840.84	22183.16
JX-2	1.11	7.04	36.78	111.57	716.5	23.24	30673.98	1819.35	24027.86
JX-3	2.02	7.02	33.73	118.09	801.84	23.02	30000.71	2222.55	20083.63
QS-1	2.15	6.54	18.17	90.95	133.66	16.93	32893.21	1455.85	4444.98
QS-2	2.13	6.54	17.7	85.65	128.56	17.68	29971.36	1092.72	4406.52
QS-3	2.67	6.81	17	80.73	125.05	17.17	31375.51	1616.57	4661.77
XC-1	1.95	6.99	123.27	137.49	786.56	44.57	28477.41	2778.88	28693.76
XC-2	1.95	7	123.76	141.36	823	43.47	29342.97	1516.52	24089.71
XC-3	1.86	7	124.23	138.74	778.02	46.12	30332.05	2321.67	24834.90

Table S4 Summary of species richness and diversity indexes for all the samples in cultivar experiment.

Sample	Seq_num	OTU_num	Chao1	ACE	Shannon	Simpson	PD_whole_tree	Fisher_alpha	Coverage
HP1	12728	1925	2761.48	2796.56	9.43	0.9964	116.48	630.42	94.19%
HP2	12728	1945	2773.85	2782.76	9.42	0.9962	116.63	639.97	94.19%
HP3	12728	1944	2892.04	2886.18	9.41	0.9963	115.73	639.49	93.90%
HS1	12728	2116	3196.03	3141.37	9.61	0.9970	123.57	724.21	93.24%
HS2	12728	2137	3037.92	3159.64	9.65	0.9972	118.30	734.87	93.38%
HS3	12728	2109	2918.01	3014.19	9.56	0.9966	123.32	720.67	93.66%
HT1	12728	2174	3274.01	3219.18	9.71	0.9972	122.34	753.83	93.10%
HT2	12728	2167	3157.31	3268.29	9.70	0.9973	124.44	750.23	93.12%
HT3	12728	2025	2948.61	2946.39	9.49	0.9965	115.85	678.80	93.77%
HZ1	12728	2129	2849.35	2888.84	9.68	0.9965	120.38	730.80	94.09%
HZ2	12728	2127	2954.56	2995.75	9.75	0.9971	121.04	729.78	93.86%
HZ3	12728	1783	2548.60	2610.82	9.05	0.9930	105.40	564.38	94.54%
MG1	12728	2033	3032.67	3030.72	9.52	0.9965	119.93	682.74	93.60%
MG2	12728	2155	3346.58	3292.27	9.55	0.9961	124.76	744.07	92.94%
MG3	12728	1889	2617.04	2685.70	9.38	0.9961	112.34	613.38	94.49%
QK1	12728	2016	2867.86	2964.52	9.47	0.9965	116.07	674.39	93.81%
QK2	12728	1894	2889.34	2877.14	9.32	0.9960	115.50	615.74	93.93%
QK3	12728	1863	2695.22	2680.76	9.34	0.9961	111.28	601.20	94.40%
TC1	12728	2116	3186.03	3168.55	9.62	0.9970	120.51	724.21	93.24%
TC2	12728	1991	3080.35	3038.50	9.42	0.9962	118.05	662.18	93.55%
TC3	12728	1989	2947.09	2926.92	9.45	0.9962	113.31	661.21	93.82%
XZ1	12728	1867	2707.31	2658.92	9.28	0.9943	113.05	603.07	94.45%
XZ2	12728	1944	2563.10	2640.31	9.50	0.9960	115.09	639.49	94.74%
XZ3	12728	1951	2682.03	2684.10	9.57	0.9969	113.26	642.85	94.56%
YT1	12728	2096	3057.80	3148.23	9.52	0.9963	123.11	714.11	93.38%
YT2	12728	2236	3366.15	3370.41	9.71	0.9968	130.56	786.11	92.81%
YT3	12728	2272	3338.72	3397.30	9.75	0.9966	131.57	805.14	92.81%
ZW1	12728	2017	2929.52	2978.42	9.55	0.9967	113.33	674.88	93.76%
ZW2	12728	2021	2827.72	2899.19	9.54	0.9966	115.18	676.84	93.95%
ZW3	12728	1956	2661.06	2738.85	9.56	0.9969	110.16	645.25	94.41%
ZY1	12728	1935	2755.87	2791.20	9.42	0.9964	114.07	635.19	94.18%
ZY2	12728	1621	2423.31	2315.93	9.21	0.9964	96.69	492.78	95.32%
ZY3	12728	1883	2765.03	2802.24	9.33	0.8859	114.3	610.56	94.19%

Table S5 Summary of species richness and diversity indexes for all samples in soil experiment.

Sample	Seq_num	OTU_num	Chao1	ACE	Shannon	Simpson	PD_whole_tree	Fisher_alpha	Coverage
CF1	14920	1321	1548.78	1579.95	8.82	0.9953	96.50	349.82	97.81%
CF2	14920	1292	1530.88	1552.33	8.76	0.9949	92.60	339.51	97.85%
CF3	14920	1327	1637.86	1676.66	8.75	0.9952	95.80	351.97	97.47%
DM1	14920	1659	2238.38	2249.22	8.70	0.9904	91.27	477.68	96.21%
DM2	14920	1817	2363.44	2372.66	9.33	0.9962	100.49	542.35	96.15%
DM3	14920	1978	2652.42	2593.50	9.52	0.9967	104.60	611.49	95.68%
HF1	14920	1151	1408.09	1382.94	8.46	0.9936	84.61	290.89	98.06%
HF2	14920	1143	1410.69	1387.13	8.35	0.9924	82.77	288.21	98.02%
HF3	14920	1081	1305.86	1316.10	8.21	0.9913	77.40	267.68	98.14%
HQ1	14920	1875	2406.21	2400.24	9.36	0.9963	111.38	566.87	96.12%
HQ2	14920	1792	2415.88	2381.03	9.00	0.9945	103.77	531.90	95.97%
HQ3	14920	1880	2646.95	2591.31	9.27	0.9957	106.38	569.01	95.55%
JX1	14920	1838	2419.09	2431.66	9.46	0.9970	107.08	551.18	96.05%
JX2	14920	1875	2402.71	2409.32	9.55	0.9969	108.32	566.87	96.21%
JX3	14920	1808	2345.19	2386.67	9.36	0.9959	100.95	538.58	96.17%
QS1	14920	1440	1775.00	1791.24	8.69	0.9923	85.97	393.22	97.29%
QS2	14920	1474	1843.36	1836.23	9.07	0.9961	87.97	405.94	97.25%
QS3	14920	1461	1866.39	1873.45	8.74	0.9927	87.15	401.06	97.07%
XC1	14920	1824	2344.27	2357.86	9.37	0.9966	102.80	545.28	96.23%
XC2	14920	1793	2271.13	2273.02	9.29	0.9961	99.11	532.32	96.40%
XC3	14920	1656	2356.01	2306.41	8.73	0.9919	93.7	476.48	95.99%

Table S6 Taxonomic compositions and relative abundances of the core OTUs identified in the cultivar and soil experiments, respectively.

Taxonomic assignment	Tomato cultivar	Soil	Soil (exclude HF and CF)
Sphingobacteriales	16.53%	3.74%	8.69%
Rhizobiales	4.58%	24.67%	11.75%
Subgroup 6	7.28%	4.46%	4.77%
Sphingomonadales	1.63%	15.37%	8.17%
Xanthomonadales	6.68%	7.10%	5.99%
Subgroup 4	7.06%	2.61%	5.66%
Nitrosomonadales	6.50%	4.04%	5.23%
Cytophagales	5.92%	0	1.86%
Burkholderiales	3.49%	1.50%	5.63%
Myxococcales	3.04%	2.68%	3.16%
TRA3-20	3.38%	0.79%	0.56%
Gemmatimonadales	3.16%	0	1.98%
Micrococcales	0.15%	7.27%	3.20%
Rhodospirillales	1.54%	3.93%	2.96%
Caulobacterales	0.44%	3.04%	0.79%
Anaerolineales	1.09%	0	3.58%
Subgroup 3	2.01%	0	1.84%
Nitrospirales	1.79%	1.48%	2.14%
Streptomycetales	0.16%	3.01%	1.35%
Others	23.59%	14.30%	20.72%

Note: The relative abundance for each bacterial order was calculated by the reads number of the core OTUs belonged to the community accounting for the total reads number of core OTUs identified in each experiment.

Table S7 Taxonomic identification of the most abudant 100 OTUs in the microbiota of tomato cultivar samples in cultivar experiment.

OUT ID	HP	HS	HT	HZ	MG	QK	TC	XZ	YT	ZW	ZY	Consensus Lineage
OTU_1	676	664	786	605	787	710	917	396	695	986	710	Bacteroidetes; Sphingobacteriales
OTU_5	637	511	290	1015	867	533	408	521	880	586	527	Verrucomicrobia; Uncultured
OTU_2	645	410	352	34	755	905	610	1	542	272	804	Bacteroidetes; Sphingobacteriales
OTU_42	540	375	245	166	525	452	402	337	632	138	506	Proteobacteria; TRA3-20
OTU_4	621	408	259	203	255	595	349	153	481	270	418	Gemmatimonadetes; Gemmatimonadales
OTU_47	190	319	468	774	185	209	326	582	314	374	207	Proteobacteria; Nitrosomonadales
OTU_13	388	380	405	147	426	407	393	149	192	348	437	Proteobacteria; Xanthomonadales
OTU_32	497	333	193	120	363	513	352	48	312	350	467	Proteobacteria; GR-WP33-30
OTU_19	58	260	152	521	295	45	276	1489	86	28	16	Proteobacteria; Methylophilales
OTU_7	420	312	164	103	264	328	302	269	263	163	272	Nitrospirae; Nitrospirales
OTU_3	224	280	347	108	259	291	310	48	170	359	253	Acidobacteria; Subgroup 4
OTU_15	309	288	251	94	161	267	273	198	207	120	303	Proteobacteria; Rhizobiales
OTU_8	94	283	273	193	277	180	199	213	268	287	174	Bacteroidetes; Sphingobacteriales
OTU_6	265	198	272	50	245	307	365	21	86	269	305	Bacteroidetes; Cytophagales
OTU_14	263	242	163	94	369	254	211	171	200	125	280	Proteobacteria; Xanthomonadales
OTU_27	373	282	215	69	192	262	242	25	200	215	276	Acidobacteria; Subgroup 6
OTU_22	216	297	338	49	234	239	252	56	132	302	213	Acidobacteria; Subgroup 4
OTU_53	254	236	165	100	162	242	233	143	152	153	241	Proteobacteria; Nitrosomonadales
OTU_34	139	197	258	196	130	130	148	307	248	124	169	Proteobacteria; Rhizobiales
OTU_137	212	192	90	150	250	202	168	163	329	86	203	Bacteroidetes; Cytophagales
OTU_59	118	257	172	133	288	160	169	162	187	252	140	Verrucomicrobia; Uncultured
OTU_9	209	185	224	32	222	257	288	23	114	207	238	Acidobacteria; Subgroup 4
OTU_10	223	190	310	74	194	169	240	35	61	207	281	Proteobacteria; Xanthomonadales
OTU_21	249	152	131	128	184	218	187	75	142	189	239	Proteobacteria
OTU_29	166	149	99	182	201	186	107	143	360	95	181	Proteobacteria; Desulfuromonadales
OTU_28	187	135	269	123	164	192	244	61	153	164	149	Proteobacteria; Sphingomonadales
OTU_74	148	142	205	97	223	156	148	43	173	272	155	Verrucomicrobia; Uncultured
OTU_76	171	160	235	96	156	143	189	62	91	196	245	Acidobacteria; Subgroup 6
OTU_689	257	181	157	41	150	196	208	35	124	151	236	Acidobacteria; Subgroup 6
OTU_11	220	197	152	54	221	211	146	55	99	168	206	Acidobacteria; Subgroup 11
OTU_30	183	154	150	16	177	338	241	11	45	151	263	Acidobacteria; Subgroup 4
OTU_45	92	126	87	208	118	122	108	246	279	137	103	Bacteroidetes; Sphingobacteriales
OTU_18	273	213	79	20	176	174	168	150	159	17	162	Proteobacteria
OTU_83	143	160	150	103	130	169	148	83	121	278	104	Acidobacteria; Subgroup 4
OTU_24	88	141	202	164	128	81	101	232	226	106	116	Bacteroidetes; Cytophagales
OTU_134	155	144	152	78	162	176	239	67	47	134	199	Proteobacteria; Burkholderiales
OTU_20	105	106	133	175	119	107	94	188	188	200	103	Verrucomicrobia; Uncultured
OTU_64	62	174	167	283	114	116	104	205	75	97	74	Proteobacteria; Burkholderiales
OTU_12	261	145	83	13	131	251	163	0	114	81	208	Proteobacteria; Nitrosomonadales
OTU_33	138	128	109	120	126	126	128	45	67	286	140	Acidobacteria; Subgroup 5
OTU_44	160	92	126	108	91	114	147	29	26	343	145	Proteobacteria; Rhodospirillales
OTU_96	214	122	65	51	228	142	114	29	192	98	117	Verrucomicrobia; Uncultured
OTU_48	148	127	108	21	181	152	173	66	156	76	123	Proteobacteria;

OTU_67	132	155	169	42	133	153	131	39	62	141	173	Acidobacteria; Subgroup 6
OTU_25	177	127	121	13	148	162	165	0	140	88	189	Acidobacteria; Subgroup 6
OTU_63	236	114	85	9	168	188	156	0	139	60	166	Proteobacteria; TRA3-20
OTU_49	58	116	226	152	71	90	82	211	120	124	70	Proteobacteria; Rhizobiales
OTU_286	68	166	143	215	79	77	112	236	60	91	57	Proteobacteria; Nitrosomonadales
OTU_62	80	108	107	218	83	75	74	150	162	133	77	Verrucomicrobia; Opitutales
OTU_36	50	93	60	423	83	41	39	211	146	42	42	Verrucomicrobia; Uncultured
OTU_214	203	126	61	17	153	163	162	1	127	54	152	Proteobacteria; TRA3-20
OTU_17	132	85	103	14	191	157	202	0	97	85	121	Bacteroidetes; Sphingobacteriales
OTU_406	81	101	148	134	78	91	139	78	102	87	144	Proteobacteria; Nitrosomonadales
OTU_39	91	85	73	111	115	123	130	88	175	73	110	Bacteroidetes; Sphingobacteriales
OTU_16	60	109	70	134	78	107	101	160	114	127	88	Cyanobacteria; D_2__Chloroplast
OTU_72	103	103	58	106	97	80	91	36	94	277	84	Verrucomicrobia; Uncultured
OTU_478	54	217	171	46	52	73	94	152	91	126	51	Acidobacteria; Subgroup 3
OTU_166	123	61	94	88	142	100	186	27	65	111	126	Proteobacteria; Xanthomonadales
OTU_340	36	223	73	82	76	40	55	272	173	71	22	Verrucomicrobia; Uncultured
OTU_117	117	73	95	64	101	96	129	40	98	97	172	Proteobacteria; Nitrosomonadales
OTU_56	56	123	137	103	109	75	67	160	99	51	91	Proteobacteria; Pseudomonadales
OTU_135	135	123	113	69	77	106	122	55	73	97	99	Proteobacteria; Nitrosomonadales
OTU_51	74	80	124	37	134	116	147	25	55	110	159	Bacteroidetes; Sphingobacteriales
OTU_106	68	114	138	76	112	105	112	59	69	88	117	Bacteroidetes; Sphingobacteriales
OTU_71	138	91	63	61	90	134	104	78	108	75	113	Acidobacteria; Uncultured
OTU_230	136	82	99	42	89	139	129	5	39	155	137	Proteobacteria; Uncultured
OTU_145	60	86	144	93	119	93	82	65	76	105	76	Bacteroidetes; Sphingobacteriales
OTU_99	118	85	63	22	128	183	139	13	69	49	128	Bacteroidetes; Cytophagales
OTU_126	53	45	131	307	40	34	56	160	121	21	26	Proteobacteria; Xanthomonadales
OTU_300	61	94	108	128	68	90	107	128	40	83	84	Proteobacteria; Burkholderiales
OTU_50	154	84	67	32	82	152	98	30	86	71	132	Acidobacteria; Uncultured
OTU_94	113	52	105	116	146	104	102	4	17	115	109	Bacteroidetes; Cytophagales
OTU_128	67	130	126	61	57	85	81	102	71	81	73	Proteobacteria; Rhodospirillales
OTU_173	95	81	95	81	61	85	63	91	85	67	109	Proteobacteria; Rhizobiales
OTU_31	114	73	27	30	109	171	102	15	143	28	96	Proteobacteria; Desulfuromonadales
OTU_26	37	70	76	169	76	59	59	102	122	90	41	Bacteroidetes; Sphingobacteriales
OTU_200	61	125	74	64	71	69	69	155	69	85	52	Acidobacteria; Subgroup 5
OTU_116	78	60	118	118	67	84	96	43	55	85	74	Bacteroidetes; Sphingobacteriales
OTU_374	6	155	118	85	49	45	28	173	140	38	12	Acidobacteria; Subgroup 4
OTU_73	102	53	78	95	91	62	95	28	76	101	51	Bacteroidetes; Sphingobacteriales
OTU_69	99	66	81	27	93	92	134	14	51	83	87	Bacteroidetes; Sphingobacteriales
OTU_54	138	47	60	45	93	118	75	20	47	45	130	Bacteroidetes; Sphingobacteriales
OTU_92	114	54	73	59	96	57	125	0	69	85	77	Proteobacteria; Xanthomonadales
OTU_776	89	50	95	96	96	87	81	8	18	99	84	Bacteroidetes; Cytophagales
OTU_231	8	49	17	305	51	11	99	246	5	7	5	Proteobacteria; Methylophilales
OTU_108	60	49	50	91	80	57	77	54	108	101	65	Bacteroidetes; Sphingobacteriales
OTU_210	47	56	95	137	43	90	84	73	55	64	43	Proteobacteria; Xanthomonadales
OTU_183	133	62	62	39	74	88	68	3	30	144	79	Acidobacteria; Subgroup 6
OTU_75	116	73	59	62	69	104	84	0	50	74	83	Proteobacteria; Nitrosomonadales
OTU_124	75	70	69	31	76	104	97	43	82	46	81	Proteobacteria; Rhodocyclales

OTU_61	1	77	135	91	36	17	24	282	87	17	1	Verrucomicrobia; OPB35 group
OTU_23	91	60	93	1	55	133	100	0	21	26	175	Proteobacteria; Myxococcales
OTU_37	163	42	34	28	40	124	72	0	47	65	122	Latescibacteria; Uncultured
OTU_245	71	97	71	41	61	88	88	70	52	47	51	Proteobacteria; Myxococcales
OTU_174	64	65	33	81	78	69	42	89	98	56	53	Verrucomicrobia; Uncultured
OTU_120	78	74	34	89	49	51	30	61	99	107	54	Verrucomicrobia; Uncultured
OTU_2617	13	50	125	92	20	93	89	140	53	31	19	Proteobacteria; Sphingomonadales
OTU_35	66	58	56	8	119	97	85	17	47	58	111	Bacteroidetes; Sphingobacteriales
OTU_144	61	48	73	148	50	31	43	61	62	69	61	Actinobacteria; Gaiellales
OTU_100	86	69	119	21	50	91	96	15	15	66	78	Proteobacteria; Burkholderiales

Note: Number of each OTU was presented as the total read number with three parallels for each treatment.

Table S8 Taxonomic identification of the most abundant 100 OTUs in the microbiota of soil samples in soil experiment.

OTU ID	CF	HF	DM	HQ	JX	QS	XC	Consensus Lineage
OTU_15	41	41	1392	922	246	732	1196	Proteobacteria; Sphingomonadales
OTU_68	0	2	684	124	1124	77	406	Bacteroidetes; Sphingobacteriales
OTU_7	60	165	302	504	591	416	193	Proteobacteria; Rhizobiales
OTU_8	0	0	7	77	47	1920	0	Acidobacteria; Subgroup 2
OTU_77	0	2	251	751	208	7	758	Chloroflexi; Anaerolineales
OTU_2	21	89	393	964	13	31	410	Actinobacteria; Micrococcales
OTU_74	43	127	417	225	560	394	105	Proteobacteria; Rhizobiales
OTU_28	70	1705	1	1	4	6	0	Proteobacteria; Methylophilales
OTU_224	34	5	350	243	456	376	237	Proteobacteria; Nitrosomonadales
OTU_128	297	203	278	382	83	31	252	Proteobacteria; Rhizobiales
OTU_9	578	882	0	2	10	0	1	Bacteroidetes; Cytophagales
OTU_17	848	292	75	10	42	1	141	Proteobacteria; Sphingomonadales
OTU_49	310	79	100	690	45	40	97	Actinobacteria; Micrococcales
OTU_42	23	0	405	475	230	28	127	Proteobacteria; Rhodospirillales
OTU_338	0	1	600	36	92	31	476	Acidobacteria; Subgroup 4
OTU_51	267	491	53	190	58	36	138	Proteobacteria; Rhizobiales
OTU_18	8	1	281	94	60	29	757	Acidobacteria; Subgroup 4
OTU_6	240	963	4	5	9	1	6	Actinobacteria; Streptosporangiales
OTU_5	742	451	4	2	2	0	1	Actinobacteria; Acidimicrobiales
OTU_25	0	1	45	483	227	347	83	Proteobacteria; Burkholderiales
OTU_1	97	1075	0	1	8	0	1	Bacteroidetes; Order II
OTU_252	7	5	361	32	496	78	181	Acidobacteria; Subgroup 6
OTU_55	58	219	169	278	109	13	309	Actinobacteria; Streptomycetales
OTU_66	188	154	372	38	176	104	119	Proteobacteria; Xanthomonadales
OTU_164	77	168	277	177	150	19	228	Proteobacteria; Xanthomonadales
OTU_148	1	0	144	112	414	264	158	Proteobacteria; Xanthomonadales
OTU_37	0	0	531	40	162	39	294	Bacteroidetes; Cytophagales
OTU_40	304	31	121	226	163	57	163	Proteobacteria; Xanthomonadales
OTU_11	401	597	4	12	4	0	9	Proteobacteria; Oceanospirillales
OTU_54	506	387	24	25	28	2	46	Proteobacteria; Rhizobiales
OTU_45	177	407	106	190	4	0	128	Bacteroidetes; Cytophagales
OTU_31	927	21	3	12	41	2	1	Proteobacteria; Xanthomonadales
OTU_10	74	901	0	1	13	1	0	Chloroflexi; Anaerolineales
OTU_97	55	28	147	572	29	2	152	Actinobacteria; Propionibacteriales
OTU_4	373	585	0	1	7	0	1	Chloroflexi; Anaerolineales
OTU_52	243	613	47	35	2	0	9	Actinobacteria; Acidimicrobiales
OTU_250	6	4	511	20	192	30	168	Acidobacteria; Subgroup 6
OTU_60	5	4	347	13	130	308	107	Proteobacteria; Rhizobiales
OTU_90	0	0	11	152	80	663	2	Acidobacteria; Acidobacteriales
OTU_136	0	0	2	17	35	853	0	Acidobacteria; Acidobacteriales
OTU_3	847	19	4	9	3	0	18	Proteobacteria; Caulobacterales
OTU_240	0	0	4	29	25	842	0	Acidobacteria; Acidobacteriales
OTU_167	6	0	152	26	385	195	135	Verrucomicrobia; Uncultured

OTU_115	0	0	3	791	51	7	7	Proteobacteria; Rhodocyclales
OTU_27	0	1	1	47	4	1	763	Proteobacteria; Burkholderiales
OTU_184	114	0	68	93	149	193	182	Nitrospirae; Nitrospirales
OTU_50	0	0	178	67	233	209	88	Nitrospirae; Nitrospirales
OTU_34	12	92	218	112	14	9	314	Bacteroidetes; Sphingobacteriales
OTU_387	0	0	211	34	117	32	369	Bacteroidetes; Sphingobacteriales
OTU_102	3	0	138	416	29	0	167	Proteobacteria; Pseudomonadales
OTU_69	272	1	40	28	4	0	400	Bacteroidetes; Sphingobacteriales
OTU_36	0	0	710	0	0	20	11	Bacteroidetes; Sphingobacteriales
OTU_238	3	0	59	294	29	99	248	Proteobacteria; Burkholderiales
OTU_76	2	18	392	108	48	31	127	Proteobacteria; Sphingomonadales
OTU_379	0	0	6	7	159	530	0	Acidobacteria; Subgroup 3
OTU_227	0	1	95	147	156	159	130	Bacteroidetes; Sphingobacteriales
OTU_125	2	678	0	3	2	0	0	Bacteroidetes; Flavobacteriales
OTU_83	0	0	266	22	276	71	40	Acidobacteria; Subgroup 11
OTU_65	0	1	112	354	21	6	176	Actinobacteria; Pseudonocardiales
OTU_73	196	454	0	0	7	0	2	Proteobacteria; Myxococcales
OTU_20	0	64	152	132	64	103	142	Proteobacteria; Sphingomonadales
OTU_2179	0	0	9	26	140	472	5	Verrucomicrobia; Uncultured
OTU_12	25	619	0	1	0	0	0	Proteobacteria; Myxococcales
OTU_108	31	36	308	4	9	6	237	Proteobacteria;
OTU_122	238	103	69	25	9	4	176	Bacteroidetes; Sphingobacteriales
OTU_757	0	0	230	99	171	40	78	Proteobacteria; GR-WP33-30
OTU_185	0	0	149	93	73	83	215	Cyanobacteria
OTU_268	4	10	48	246	138	42	122	Proteobacteria; Burkholderiales
OTU_138	6	0	78	125	115	175	109	Verrucomicrobia; Opitutales
OTU_149	27	9	198	226	80	18	47	Chloroflexi; KD4-96
OTU_82	0	0	75	30	25	14	454	Gemmatimonadetes; Gemmatimonadales
OTU_71	18	24	41	92	17	104	299	Proteobacteria; Pseudomonadales
OTU_477	229	362	0	0	0	0	0	Gemmatimonadetes; Gemmatimonadales
OTU_29	0	0	154	4	406	5	22	Latescibacteria; Uncultured
OTU_92	0	0	368	18	169	8	25	Gemmatimonadetes; Gemmatimonadales
OTU_207	154	281	30	88	8	0	24	Proteobacteria; Rhizobiales
OTU_179	1	0	89	63	100	229	101	Proteobacteria; Nitrosomonadales
OTU_35	473	14	16	20	28	0	29	Proteobacteria; Myxococcales
OTU_346	0	0	68	107	32	57	310	Proteobacteria; Myxococcales
OTU_415	8	2	125	113	159	139	27	Proteobacteria; Rhodospirillales
OTU_32	156	389	10	1	4	0	13	Proteobacteria; Myxococcales
OTU_604	26	22	65	127	85	142	92	Proteobacteria; Myxococcales
OTU_134	4	0	99	62	220	85	88	Proteobacteria; Nitrosomonadales
OTU_21	158	388	0	0	5	0	0	Chloroflexi; Anaerolineales
OTU_46	546	2	0	0	2	0	0	Saccharibacteria; Uncultured
OTU_211	171	66	80	69	42	68	54	Proteobacteria; Rhizobiales
OTU_944	45	17	83	167	105	69	60	Actinobacteria
OTU_56	0	0	5	0	11	527	0	Acidobacteria; Subgroup 2
OTU_14	9	526	0	0	4	0	0	Proteobacteria; Methylophilales
OTU_220	52	81	11	9	57	312	15	Proteobacteria; Rhizobiales

OTU_62	8	1	56	49	145	5	268	Proteobacteria; Sphingomonadales
OTU_178	379	111	1	34	2	1	3	Proteobacteria; Rhizobiales
OTU_13	521	5	0	0	1	0	0	Bacteroidetes; Sphingobacteriales
OTU_23	64	458	0	0	1	0	0	Proteobacteria; Oceanospirillales
OTU_30	39	11	89	162	123	29	70	Cyanobacteria
OTU_78	1	1	197	3	269	21	28	Nitrospirae; Nitrospirales
OTU_16	515	1	0	0	0	0	0	Chloroflexi; Anaerolineales
OTU_22	185	320	0	0	0	0	0	TM6; Uncultured
OTU_174	0	0	156	5	76	120	145	Bacteroidetes; Sphingobacteriales
OTU_132	0	0	0	9	89	402	0	Bacteroidetes; Cytophagales

Note: Number of each OTU was presented as the total read number with three parallels for each treatment.

Fig. S1 The composition and relative abundance of the major bacterial taxa in the tomato rhizosphere microbiotas in cultivar experiment. Each bar represents the average value of three replicates in each sample group. A. The composition and relative abundance of major bacterial phyla; B-D. The composition and relative abundance of the major bacterial orders from the phyla of Bacteriodetes (B), Acidobacteria (C) and Proteobacteria (D); E. The composition and relative abundance of the major bacterial orders from four classes of the phylum Proteobacteria: Alpha-, Beta-, Delta- and Gamma-proteobacteria. Xinzhongshu No. 4 (XZ), Huangshoutao (HT), Tiancheng (TC), Meiguodahong 168 (MG), Huapiqiu (HP), Huangshengnvguo (HS), Huangzhenzhu (HZ), Qiaokeli (QK), Yingtao (YT), Ziwucai (ZW) and Ziyixiannv (ZY).

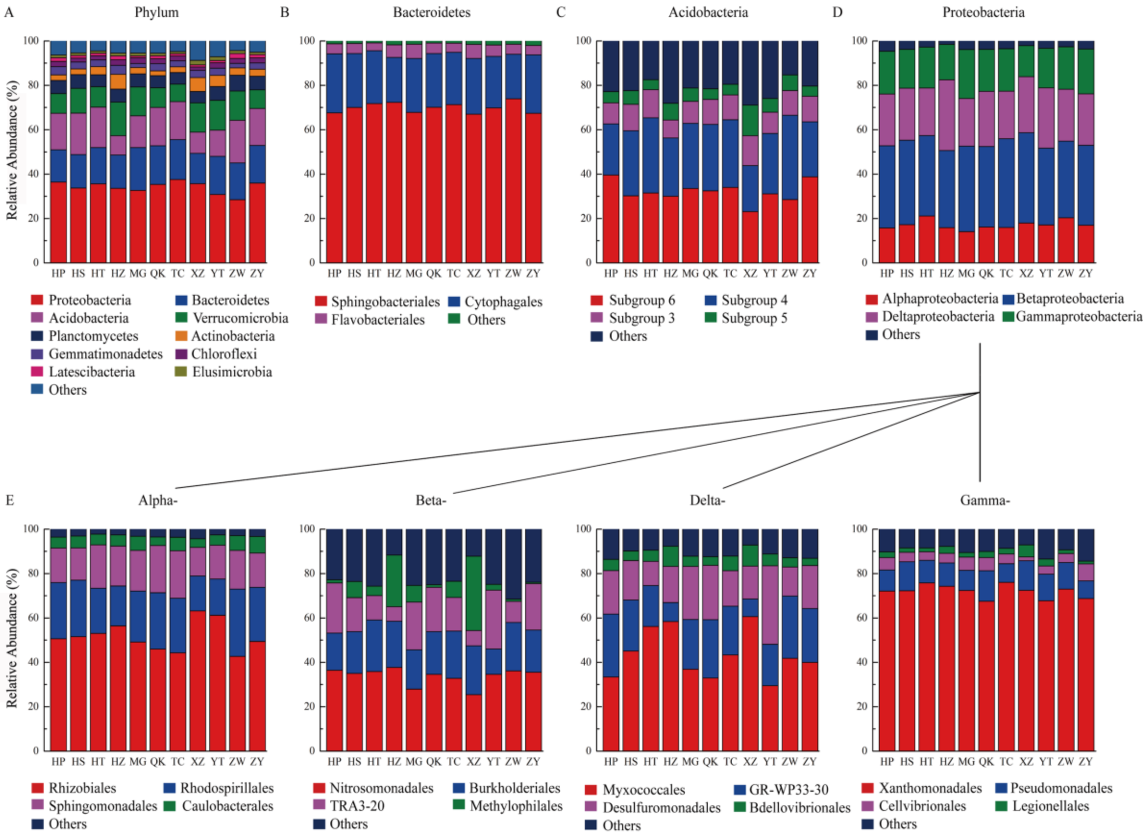

Fig. S2 The percentage of core OTUs to be presented in the samples. A: Tomato cultivar experiment; B: Soil experiment; C: soil experiment except for samples CF and HF.

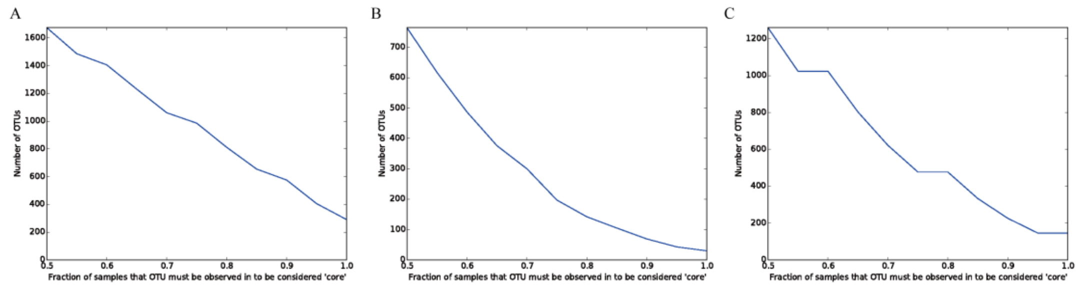

Fig. S3 The OTU number boxplots and the rank abundance curves indicate OTU richness of the sample in the cultivar and soil experiments, respectively. The plots were drawn using the average value of three replicates of each sample group. A. The OTU number boxplot depicting OTU richness in the microbiota of tomato cultivar samples; B. The rank-abundance curve depicting species richness and evenness in the microbiota of tomato cultivar samples; C. The OTU number boxplot depicting OTU richness in the microbiota of soil samples; D. The rank-abundance curve depicting species richness and evenness in the microbiota of soil samples. Tomato cultivar experiment: Xinzhongshu No. 4 (XZ), Huangshoutao (HT), Tiancheng (TC), Meiguodahong 168 (MG), Huapiqiu (HP), Huangshengnvguo (HS), Huangzhenzhu (HZ), Qiaokeli (QK), Yingtao (YT), Ziwucai (ZW) and Ziyixiannv (ZY); soil experiment: commodity organic nutritional soil (CF), potted plant nutrients soil (HF), vegetable field soil (DM), agricultural field soil (HQ), campus lawn (JX), forest soil (QS), garden soil (XC).

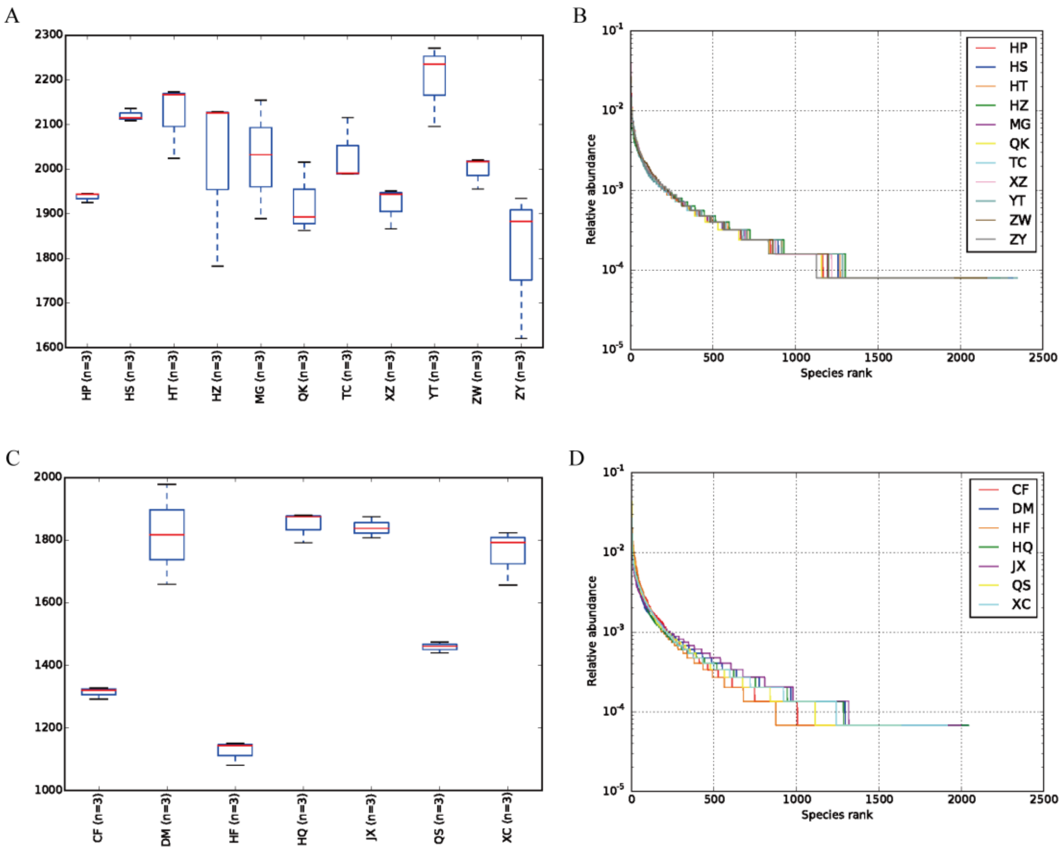

Fig. S4 Rarefaction curves and the OTU number boxplot of the rhizosphere microbiota in the cultivar or soil experiments. A. Rarefaction curves of the different tomato cultivar samples in cultivar experiment; B. Rarefaction curves of the different sources of soil samples in soil experiment; C. Rarefaction curves of all samples in both the cultivar and soil experiments; D. The OTU number boxplot of the samples in both the cultivar and soil experiments.

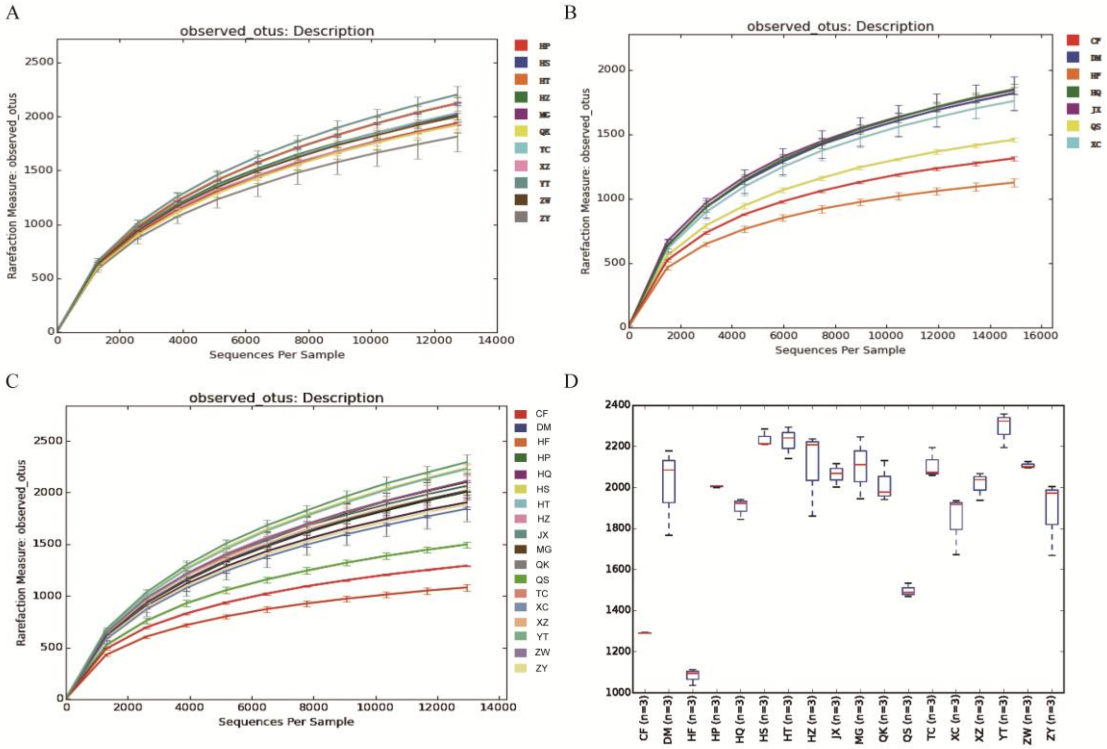

Fig. S5 The composition and relative abundance of the major bacterial taxa in soil samples in soil experiment. Each bar represents the average value of three replicates in each sample group. A. The composition and relative abundance of the major bacterial phyla; B-D. The composition and relative abundance of the major bacterial orders from the phyla of Bacteriodetes (B), Acidobacteria (C) and Proteobacteria (D); E. The composition and relative abundance of the major bacterial orders from four classes of the phylum Proteobacteria: Alpha-proteobacteria, Beta-proteobacteria, Delta-Proteobacteria and Gamma-proteobacteria. Commodity organic nutritional soil (CF), potted plant nutrients soil (HF), vegetable field soil (DM), agricultural field soil (HQ), campus lawn (JX), forest soil (QS) and garden soil (XC).

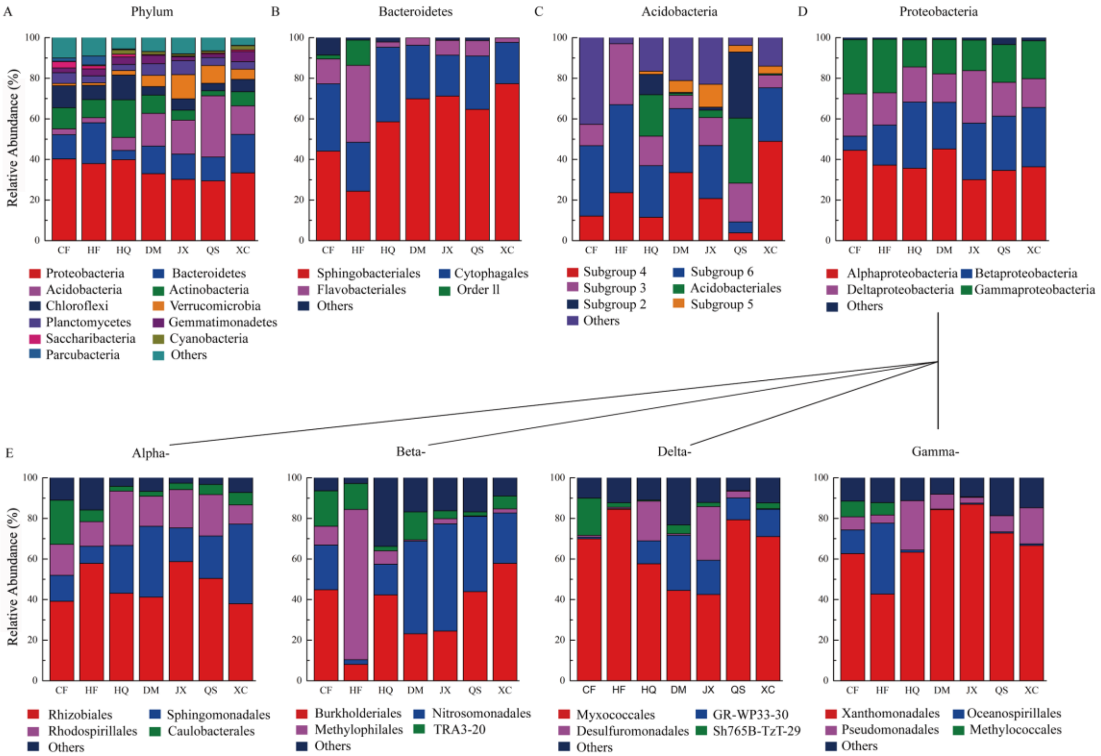

Fig. S6 Beta diversity analysis to estimate the dissimilarity and similarity of bacterial communities and composition among the samples. A. Principal coordinated analysis (PCoA) derived from the dissimilarity matrix of the weighted UniFrac distance among the cultivar samples in cultivar experiment. B. The hierarchical cluster analysis of the bacterial community composition among the cultivar samples in cultivar experiment. C. Principal coordinated analysis (PCoA) derived from the dissimilarity matrix of the weighted UniFrac distance among the soil samples in soil experiment. D. The hierarchical cluster analysis of the bacterial community composition among the soil samples in soil experiment.

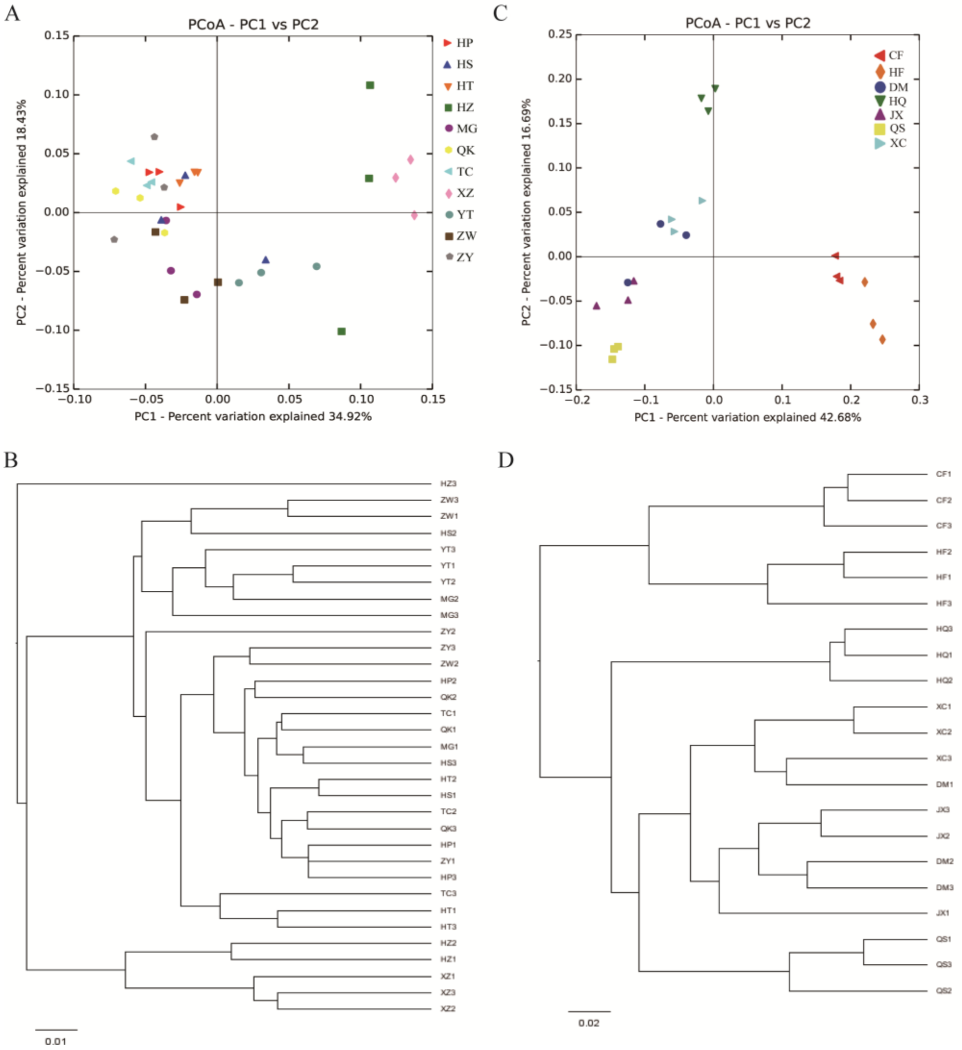

Supplement: Supplementary file 1 [file microorganisms-08-00170-s001.pdf]
